# Metastatic renal clear cell carcinoma mimicking a gallbladder polyp: Case report and literature review

**DOI:** 10.1016/j.ijscr.2019.09.032

**Published:** 2019-09-25

**Authors:** Skander Zouari, Mouna Ben Othmen, Nihed Abdessayed, Nadia Larbi Mama, Mohamed Salah Jarrar, Badreddine Sriha, Moncef Mokni, Mehdi Jaidane, Wissem Hmida

**Affiliations:** a Sahloul Hospital, Department of Urology, Route de la ceinture, Hammam Sousse, 4011, Sousse, Tunisia; b Farhat Hached Hospital, Department of Histopathology, Ibn El Jazzar Road, Sousse Ezzouhour, 4031, Sousse, Tunisia; c Research Lab: Transfer in Technology in Anatomic Pathology (LR12SP08), Tunisia; d Sahloul Hospital, Department of Radiology, Route de la ceinture, Hammam Sousse, 4011, Sousse, Tunisia; e Farhat Hached Hospital, Department of General Surgery, Ibn El Jazzar Road, Sousse Ezzouhour, 4031, Sousse, Tunisia

**Keywords:** Renal cell carcinoma, Gallbladder, Cancer, Metastasis, Polyp, Case report

## Abstract

**Introduction:**

Metastatic tumors to the gallbladder are uncommon. Metastases from renal cell carcinoma (RCC) to the gallbladder are exceptional. Frequencies of less than 0.6% reported in large autopsy reviews and few cases have been reported in the literature.

**Presentation of case:**

Herein we present a case of a 50-year-old man that developed four years after radical nephrectomy for RCC, a gallbladder metastasis, discovered incidentally on Computed Tomography (CT) scan. It was described as an intraluminal gallbladder polyp. Radiological features were very suggestive of primary gallbladder carcinoma, thus the patient had a laparoscopic cholecystectomy. The pathological examination of the surgical specimen concluded to a RCC metastasis. Immunochemistry with vimentin and pancytokeratin were supportive of this diagnosis. The clinical course was uneventful after 18 months period of follow up.

**Discussion:**

At the time of presentation, almost one-third of the patients with RCC are metastatic. Metastases to the gallbladder are extremely rare. Clinical presentation and physical examination are unspecific. Radiological findings can raise information and orient the diagnosis although the difference between both diagnoses remains difficult. Usually, it is a pedunculated and not associated with gallstones with enhancement on CT scan. The treatment remains surgical with R0 cholecystectomy, and prognosis is mainly related to disease free interval and single site metastasis.

**Conclusion:**

We highlight here the challenge to make the difference between a primary gallbladder carcinoma and metastasis from RCC. Diagnosis is made on pathological examination and immunochemistry.

## Highlights

• Metastases to gallbladder from renal cell carcinoma are very rare.
• Most of the cases are diagnosed incidentally, and both clinical presentation and physical examination are unspecific.
• Imaging although its specificity can’t make the difference between primary gallbladder carcinoma and metastasis from RCC.
• Only pathological examination of the specimen after cholecytectomy with immunochemistery can assess the diagnosis.

## 1 Introduction

Renal cell carcinoma (RCC) is a rare tumor accounting for 3% of all malignancies in adults [1]. This tumor has a great propensity to metastasize synchronously or metachronously to various anatomic sites [2]. The metastatic locations of an RCC are mainly lungs, bones, lymph nodes, liver, and adrenal glands. Gallbladder remains an extremely rare metastatic site. To our knowledge, only 58 cases of gallbladder metastasis from RCC have been reported so far. Herein we report a case of intraluminal polypoid metastasis of clear RCC in gallbladder mimicking gallbladder polyp. Clinic-pathological features will be discussed emphasizing differential diagnosis with a literature review. The work has been reported in line with the SCARE criteria [3].

## 2 Case presentation

A 50-year-old man underwent 5 years ago a right nephrectomy for renal tumor diagnosed on the basis of total hematuria and Computed Tomography (CT) scan imaging. The pathological examination that time, concluded to a conventional clear cell RCC, Fuhrman nuclear grade 3. The tumor invaded the right renal vein, without other metastatic locations. All margins were free of tumor and it was staged pT3N0M0. The patient didn’t receive adjuvant chemotherapy after the surgery and was followed closely.

**Fig. 1:**
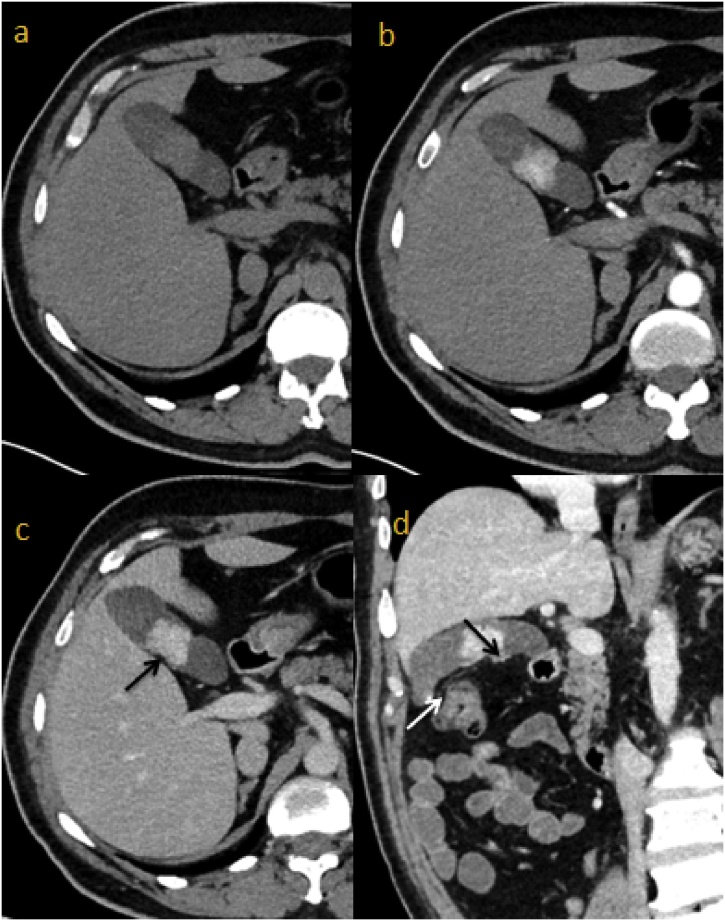
Abdominal thin slices CT scan in axial plan : non enhanced (a), arterial (b) and portal acquisition (c). Coronal oblique thin slice CT scan (d): Lobulated density mass, in the gallbladder, with intense arterial enhancement after contrast injection, and retracting gallbladder wall (black arrows). It was associated with gallstones (white arrow).

Four years after the first diagnosis and during follow up, a CT scan showed a tissue density mass in the gallbladder, measuring 30 × 31 mm, with lobulated contours retracting the gallbladder wall. An intense arterial enhancement after contrast injection was noted. Intraluminal gallstones were found (Fig. 1). Clinically, the patient was asymptomatic. On physical exam, neither abdominal tenderness nor icterus was noted. Laboratory exams didn’t show biological cholestasis (total bilirubin 0.5 mg/dL, direct bilirubin 0.1 mg/dL, aspartate transaminase 16 IU/L and alanine transaminase 11 IU/L). Despite the lack of biliary symptoms and based on the radiologic appearance of the tumor on CT scan, we suggested a possible diagnosis of gallbladder primary carcinoma. A laparoscopic cholecystectomy was performed. The postoperative course was uneventful and the patient was discharged two days after the operation.

**Fig. 2:**
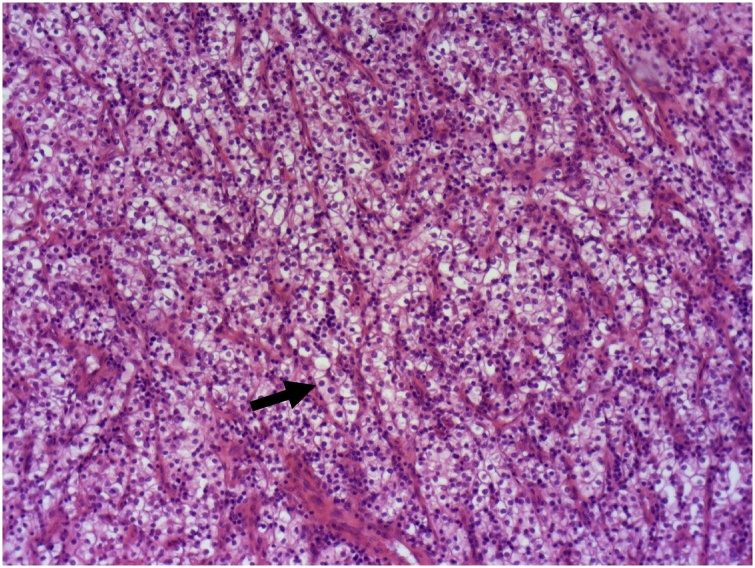
The polyp is consisted of clear cells sheets (↗), nests and cords separated by a delicate capillary vascular network (HEx50).

**Fig. 3:**
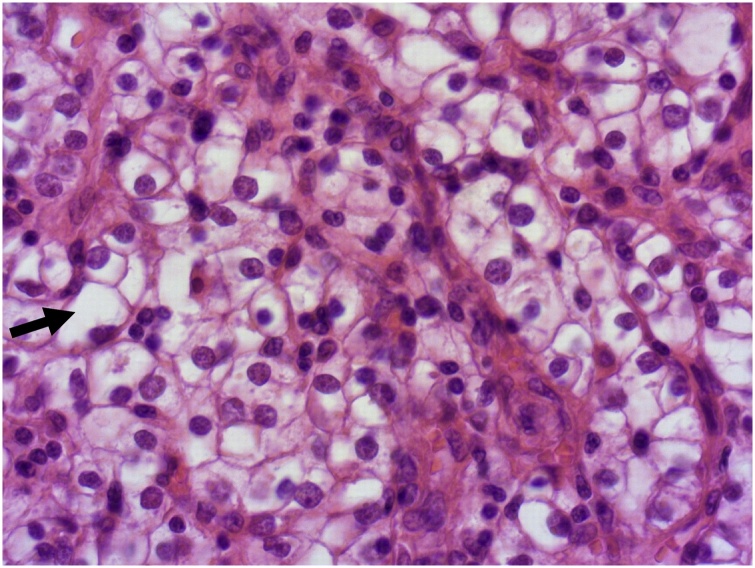
Details of tumor cells with an abundant clear cytoplasm (↗) and a round uniform nucleus (HEx200).

**Fig. 4:**
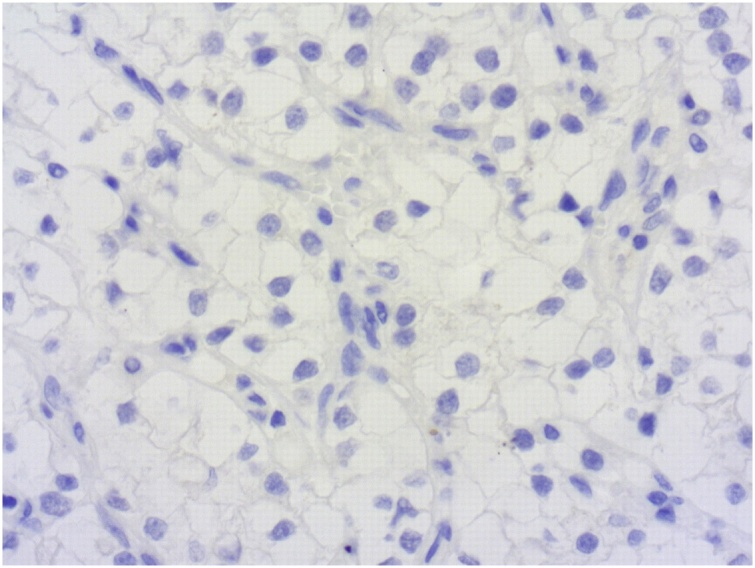
Negative staining for cytokeratin (IHCx200).

**Fig. 5:**
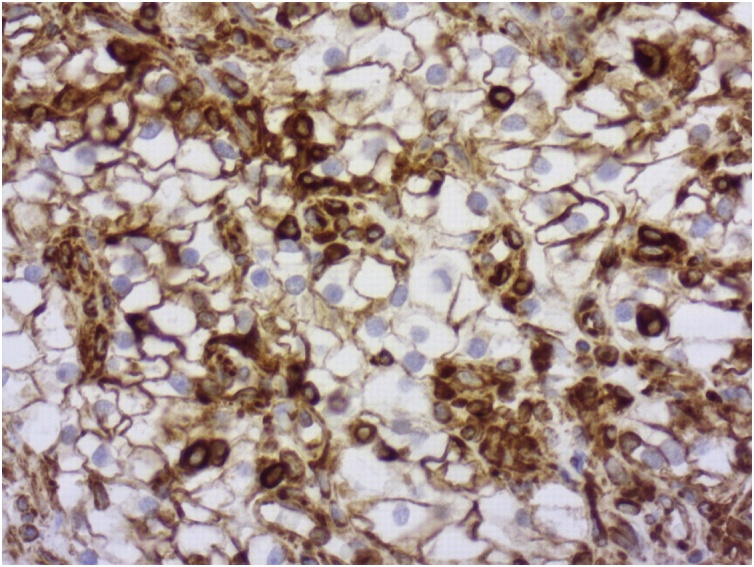
Positive and diffuse staining for vimentin (IHCx200).

The cholecystectomy specimen had a 3 × 2.5 cm polypoid mass protruding in the lumen located 4 cm from the fundus of the gallbladder. Cut surface of the mass was homogenous, yellowish and soft, with foci of hemorrhage. Microscopically, the polyp consisted of clear cell sheets, nests, and cords separated with a delicate capillary vascular network (Fig. 2). Some areas of solid and alveolar pattern were seen. Tumor cells had an abundant, clear cytoplasm surrounded by a distinct cell membrane. The nucleus was round and uniform (Fig. 3). The diagnosis of metastatic RCC was confirmed by immunohistochemical stains, which showed strong positivity for vimentin and PAX8, patchy positivity for CD10 and negativity of pancytokeratin (Fig. 4, Fig. 5). The tumor involved mucosa with no invasion of the gallbladder wall. The cystic duct margin was free of tumor.

After 18 months period of regular follow up, CT scan showed no recurrence and the patient is free from disease.

## 3 Discussion

RCC represents around 5% and 3% of all malignancies, respectively in men and women [4]. At the time of presentation, almost one-third of the patients are metastatic. Thereabouts 25 to 50% of the patients will develop metastases after radical nephrectomy [5]. The most common metastatic sites are lungs, bones, lymph nodes, liver and brain [6]. Metastases to the gallbladder from RCC are extremely rare with a 0.58% frequencies reported in large autopsy reviews [7]. Malignant melanomas are the first cause of metastasis in the gallbladder, followed by other digestive system carcinomas [8]. The pathogenesis of metastasis development in the gallbladder is explained by authors as a process whether resulting from direct invasion or from the blood spread of tumor cells secondary to vascular invasion [9] and it is more likely to raise metachronously. Based on a systematic Pubmed search using the keywords “gallbladder metastasis renal cell carcinoma”, we found 58 published cases in the literature. All the cases are summarized in (Table 1). The data has been analyzed using SPSS version 19.0 software. The main limitation of our analysis is the lack of information in some cases.

**Table 1:** Reported cases of gallbladder metastasis from renal cell carcinoma.

Author	Sex	Age at time of diagnosis RCC (years)	Time from RCC (years)	Other metastasis sites	Symptom	Imaging findings/Intraoperative findings			Follow up (months)	Recurrence	Adjuvant therapy
						Pedunculated	Gallstone	Contrast enhancement			
Harder VV (1983)	M	N/A	N/A	No	N/A	N/A	N/A	N/A	N/A	N/A	N/A
Guerreiro González R (1994)	M	68	syn	No	None	No	No	No	N/A	N/A	N/A
Coşkun F (1995)	M	52	syn	No	None	Yes	N/A	N/A	N/A	N/A	N/A
Pagano S (1995)	M	62	syn	Lung	None	Yes	Yes	N/A	36	No	N/A
King DH (1995)	M	64	syn	No	None	Yes	No	N/A	26	No	N/A
Finkelstein LH (1996)	M	75	5	No	Abdominal pain	No	No	No	N/A	N/A	N/A
Lombardo FP (1996)	M	77	5	No	Abdominal pain	Yes	Yes	Yes	N/A	N/A	N/A
Uchiyama T (1997)	M	N/A	N/A	N/A	N/A	N/A	N/A	N/A	N/A	N/A	N/A
Sparwasser C (1997)	M	46	4	No	Abdominal pain	Yes	No	N/A	12 (death)	Yes (Controlateral kidney, Adrenal gland, bone)	N/A
Celebi I (1998)	M	73	Syn	Lung	Hematuria^a^	Yes	No	N/A	1 (death)	N/A	N/A
Brasseur P (1999)	M	N/A	N/A	N/A	N/A	N/A	N/A	N/A	N/A	N/A	N/A
Kechrid M (2000)	F	55	8	Controlateral kidney, Adrenal gland	Right loin pain nausea, weight loss^a^	Yes	N/A	N/A	32 (death)	N/A	No
Hisa T (2001)	M	73	8	No	None	Yes	No	Yes	96	No	No
Aoki T (2002)	M	63	27	No	None	Yes	No	Yes	N/A	N/A	N/A
	M	80	8	No	None	Yes	No	Yes	N/A	N/A	N/A
Park JS (2003)	M	48	3	Scalp	None	Yes	No	N/A	No	No	Interleukin 2
Limani K (2003)	M	64	1	No	None	Yes	Yes	Yes	N/A	N/A	N/A
Ishizawa T (2006)	M	73	5	No	None	Yes	No	Yes	24	No	No
Pandey D (2006)	M	46	1	No	None	N/A	N/A	N/A	16	No	No
Nojima H (2008)	M	63	syn	No	Abdominal pain	Yes	No	Yes	10	No	No
Ricci V (2008)	F	58	Syn	Pancreas	N/A	Yes	N/A	N/A	N/A	N/A	N/A
Moujahid M (2008)	M	56	Syn	No	Abdominal pain	N/A	N/A	N/A	8	No	No
Sand M (2009)	F	43	5	No	Adbominal pain	No	No	No	4	No	No
Kücükakin B (2009)	M	80	2	No	Acute biliary symptom Hematuria^a^	Yes	N/A	N/A	N/A	N/A	N/A
Patel S (2009)	F	58	6	No	Abdominal pain	Yes	Yes	N/A	N/A	N/A	No
Shoji S (2010)	M	47	3	No	No	Yes	No	Yes	8	No	No
Fang X (2010)	M	45	1	Lung	No	Yes	No	Yes	28	Lung	Chemoradiation
	F	65	2	Left Psoas/Right neural foramina	Abdominal pain	Yes	No	Yes	7 (death)	Bone	No
	M	54	7	No	No	Yes	No	N/A	27	No	No
	M	51	7	No	No	Yes	No	N/A	37	Retroperitoneum/Pancreas	Chemotherapy
Kawahara T (2010)	F	73	Syn	Lung	Hematuria^a^	Yes	No	N/A	N/A	N/A	N/A
Decoene J (2011)	F	47	16	Left Calcaneus	None	Yes	No	N/A	N/A	N/A	N/A
Chung PH (2012)	F	53	10	No	N/A	Yes	N/A	N/A	84	Controlateral kidney, lung, bone, pancreas	N/A
	F	52	Syn	No	N/A	Yes	N/A	N/A	60	Controlateral kidney, lung, bone, pancreas	N/A
	M	51	Syn	Lung, bone, controlateral kidney	N/A	Yes	N/A	N/A	132 (death)	Ethmoid, orbit, cribriform	N/A
	M	42	Syn	No	N/A	Yes	N/A	N/A	6 (death)	Controlateral Kidney, spine	N/A
Robledo AB (2012)	F	75	1	Adrenal gland	None	Yes	No	N/A	4	No	No
Zygulska AL (2012)	F	70	3	No	None	Yes	Yes	N/A	1	Pancreas	Pancreatectomy
Collin Y (2012)	M	68	1	No	Abdominal Pain	No	No	N/A	N/A	N/A	N/A
Seeliger B (2013)	M	44	2	No	Abdominal pain	Yes	N/A	N/A	22 (death)	Brain, liver, skin	Interferon/Radiotherapy
	F	83	1	No	None	Yes	No	Yes	55	No	No
Jain D (2013)	F	49	6	No	Abdominal pain	Yes	Yes	N/A	N/A	N/A	N/A
Zevallos Quiroz JC (2014)	F	55	6	Controlateral Kidney	None	Yes	No	Yes	N/A	N/A	N/A
Turner G (2014)	F	55	8	No	Abdominal Pain	N/A	N/A	N/A	N/A	N/A	N/A
Win AZ. (2014)	M	40	21	No	Abdominal Pain	Yes	No	Yes	N/A	N/A	N/A
Ueda I (2015)	M	42	1	No	None	Yes	Yes	Yes	N/A	N/A	N/A
Castro Ruiz C (2015)	M	48	3	Lung	None	No	No	Yes	7	No	No
Costa Neves M (2016)	F	60	2	No	None	Yes	No	No	2	No	No
	M	57	1	No	Abdominal pain	Yes	No	NA	38	No	No
Kamido S (2016)	M	57	6	No	None	Yes	No	Yes	N/A	N/A	N/A
Mrak K (2016)	M	64	12	No	None	Yes	No	Yes	N/A	N/A	N/A
Shyr (2017)	M	66	14	No	None	Yes	No	Yes	42	No	No
Saito Y (2018)	F	60	15	No	None	Yes	No	Yes	36	No	No
Kitamura H (2018)	M	62	6	No	None	Yes	No	Yes	5	Gastric/Lung	Sunitinib
Takenaka M (2018)	M	72	N/A	No	Abdominal pain	Yes	No	Yes	N/A	N/A	N/A
Takagi K (2019)	F	55	N/A	No	None	Yes	No	Yes	9	No	No
Kinoshita O (2019)	M	60	3	Gastric	None	Yes	No	Yes	12	No	No
Alves Ribeiro M (2019)	M	74	9	No	None	Yes	No	Yes	12	Pancreas	Radiotherapy
*Present case*	**M**	**45**	**5**	**No**	**None**	**Yes**	**Yes**	**Yes**	**18**	**No**	**No**

N/A, not available; Syn, synchronous.

Keyword search: gallbladder metastasis renal cell carcinoma.

a No symptom due to gallbladder metastasis.

Analysis of the data collected showed that 69.5% of the patients were male while 30.5% were female. The RCC occurred at a mean age of 58 years. The free median interval disease was 4.81 years. These results are similar to those found in the literature [10]. At the time of diagnosis, the gallbladder was the unique site of metastasis in 75.4%. When other metachronous metastases are diagnosed, the main sites are lungs 8.8% or multiple (more than 3) in 5.3%. Other sites are pancreas, contralateral kidney, adrenal gland, scalp, and stomach. Most of the time, the diagnosis is made incidentally on CT scan, with an incidence of 63.5% according to our review. The most frequent symptoms in the clinical presentation are abdominal pain (28.8%), haematuria (3.4%), nausea and vomiting (1.7%). The physical examination is generally poor. When the metastasis is synchronous with the RCC, symptoms may be related to the primary tumor. Clinically, the challenge is to make the differential diagnosis between primary gallbladder carcinoma and metastasis from RCC. Though its rarity, the primary gallbladder carcinoma incidence is higher. That’s why in about half the cases according to the literature like the present case, there is a misdiagnosis and the final diagnosis is carried out after the final pathological examination.

Radiological findings can raise information and orient the diagnosis although the difference between both diagnoses remains difficult. In its literature review, Neves et al. [10], find that lesion on CT scan is more likely to be played/pedunculated and not associated with gallstones. These findings are in accordance with our data as we found that the lesion is described as popped in 90.6% while the association with gallstones is in only 18.2% of the cases. Kitamura et al. [11], described a hyper-vascular and strongly enhanced lesion on CT scan that suggests metastasis from RCC whereas primary gallbladder cancer does not present such radiological patterns. Strong enhancement of the lesion was found in our review in 86.2% of the cases. But these radiological features that describe the metastasis are not always present. In our case, the tumor was described as a density mass in the gallbladder, with lobulated contours retracting the gallbladder wall. An intense arterial enhancement after contrast injection was noticed. Gallstones were also found.

The final diagnosis is made on specimen pathological examination. Macroscopically, it is generally a well-circumscribed polypoid mass with a narrow stalk located in the fundus. Neves [10] described it as a pedunculated mass with hemorrhage in the protruded portion. Low and high power fields reveal a prominent vascular proliferation as well as tumor cells with a clear cytoplasm along with vascular interstitial tissue that reminds the features of a renal primary tumor. This proliferation develops under the mucosal epithelial layer of the gallbladder. The surface is covered by gallbladder epithelium. Other lesions like adenomyomatosis or chronic cholecystitis may be associated [12]. Immunohistochemical staining is performed to assess the diagnosis and shows positivity to AE1/AE3, Vimentin, CD10 and RCC with no expression of S100, cytokeratin or CEA.

Either way, surgical treatment is required, and an R0 resection laparoscopic cholecystectomy is performed as the tumor is generally developed with the mucosa. When there is a doubt of locally advanced tumors, a frozen section can be performed to check the serosal involvement. When metastasectomy is achievable, surgery must be done to prolong overall and cancer-specific survival [11], especially with favorable disease factors as a solitary metastasis, and a long free disease interval.

Recent literature reviews [9,10,13], found more than half of those patients alive with no evidence of disease, despite the short follow up. According to our review, we identified 34 cases with follow up included. The mean follows up time was 26.94 months, with recurrence occurring in 35.3% of the cases. Most of the recurrences were multiple in 66.7%. Other recurrences involved pancreas 16.7%, bones 8.3% or lungs 8.3%. The death occurred in 6 cases (17.1%). Some authors tried to identify prognosis factors in their multivariate analysis: Shyr et al. [14] demonstrated that time from initial diagnosis of the RCC was the only favorable predictor of survival with 5 years survival estimated to 59% whereas Kavoluis [15] found that both disease-free interval and single site metastasis are prognosis factors.

The use of chemotherapy (n = 2), radiation therapy (n = 1) or antiangiogenic (n = 1) was described in some cases as an adjuvant treatment, without concrete evidence for their role after metastasectomy. Several phases 3 studies are on their ways to investigate their place in adjuvant treatment [10].

## 4 Conclusion

Metastasis to the gallbladder from an RCC is an uncommon entity. We must keep in mind the diagnosis when a tumor is detected in the gallbladder by the US or CT scan, mostly incidentally. The difference between primary gallbladder carcinoma and metastasis from RCC is difficult, and the final diagnosis is often made on pathological examination. R0 cholecystectomy is required, especially when it is a single metastasis, to provide remission and increase survival.

## Funding

This research did not receive any specific grant from funding agencies in the public, commercial, or not-for-profit sectors.

## Ethical approval

Given the nature of the article, a case report, no ethical approval was required.

## Consent

Written informed consent was obtained from the patient for publication of this case and accompanying images. A copy of the written consent is available for review by the Editor-in-Chief of this journal on request.

## Author’s contribution

- Skander Zouari: Writing - original draft.
- Mouna Ben Othmen: Writing - review & editing.
- Nihed Abdessaeid: Histopathological examination of the specimen and reporting, data collection.
- Nadia Mama Larbi: Data interpretation of the radiological findings.
- Mohamed Salah Jarrar: Data collection.
- Badreddine Sriha: Study concept and design, data collection.
- Moncef Mokni: Data collection, Editing of the manuscript.
- Mehdi Jaidane: Supervision; Reviewing and editing.
- Wissem Hmida: Project administration.

## Registration of research studies

This does not apply as it is a case report of a patient who has given written consent and has been de-identified. It is therefore not prospective research involving human participant.

## Guarantor

Dr. Nihed Abdessayed.

## Provenance and peer review

Not commissioned, externally peer-reviewed.

## Declaration of Competing Interest

The authors have no conflict of interest to declare.

## References

[1] McLaughlin J.K., Blot W.J., Devesa S.S., Fraumeni J.F., Schottenfeld D., Fraumeni J.F. (1996). Renal cancer. Cancer Epidemiology and Prevention.

[2] Fang X., Gupta N., Shen S.S., Tamboli P., Charnsangavej C., Rashid A., Wang H. (2010). Intraluminal polypoid metastasis of renal cell carcinoma in gallbladder mimicking gallbladder polyp. Arch. Pathol. Lab. Med..

[3] Agha R.A., Borrelli M.R., Farwana R., Koshy K., Fowler A., Orgill D.P., For the SCARE Group (2018). The SCARE 2018 statement: updating consensus Surgical CAse REport (SCARE) guidelines. Int. J. Surg..

[4] Siegel R.L., Miller K.D., Jemal A. (2018). Cancer statistics, 2018. CA Cancer J. Clin..

[5] Sand M., Bechara F.G., Kopp J. (2009). Gallbladder metastasis from renal cell carcinoma mimicking acute cholecystitis. Eur. J. Med. Res..

[6] Maldazys J.D., DeKernion J.B. (1986). Prognostic factors in metastatic renal carcinoma. J. Urol..

[7] Nojima H., Cho A., Yamamoto H., Nagata M., Takiguchi N., Kainuma O., Souda H., Gunji H., Miyazaki A., Ikeda A., Matsumoto I., Asano T., Ryu M., Nihei N., Maruoka M. (2008). Renal cell carcinoma with unusual metastasis to the gallbladder. J. Hepato-Biliary-Pancreat. Surg..

[8] Ueda I., Aoki T., Oki H. (2015). Gallbladder metastasis from renal cell carcinoma: a case report with review of the literature. Magn. Reson. Med. Sci..

[9] Saito Yasufumi, Okuda Hiroshi, Yoshida Makoto, Okimasa Seiji, Fukuda Toshikatsu, Yano Masatsugu, Ochi Makoto, Okamoto Yuzo, Nakayama Hirofumi, Ono Eiji, Ohdan Hideki (2018). Gallbladder metastasis of renal clear cell carcinoma 15 years after primary cancer excision: a case report. J. Med. Case Rep..

[10] Neves M.C., Neofytou K., Giakoustidis A., Hazell S., Wotherspoon A., Gore M., Mudan S. (2016). Two cases of gallbladder metastasis from renal cell carcinoma and review of literature. World J. Surg. Oncol..

[11] Kitamura Hirotaka, Kurokawa Masaru, Inaki Noriyuki, Bando Hiroyuki (2017). Gallbladder metastasis from renal cell carcinoma. Indian J. Surg..

[12] Castro Ruiz C., Pedrazzoli C., Bonacini S. (2016). Gallbladder’s clear cell renal carcinoma metastasis: a case report. Int. J. Surg. Case Rep..

[13] Takagi Kimiaki, Kawase Kota, Minoshima Kenichi, Yamaha Masayoshi, Maekawa Yuka, Yokoi Shigeaki, Kusakabe Mitsuhiko (2019).

[14] Shyr B.U., Chen S.C., Shyr Y.M., Lee R.C., Wang S.E. (2017). Metastatic polyp of the gallbladder from renal cell carcinoma. BMC Cancer.

[15] Kavolius J.P., Mastorakos D.P., Pavlovich C., Russo P., Burt M.E., Brady M.S. (1998). Resection of metastatic renal cell carcinoma. J. Clin. Oncol..

